# Sorghum surpasses wheat as a feed grain for broiler chickens following dietary crude protein reductions

**DOI:** 10.1186/s40104-024-01147-9

**Published:** 2025-02-06

**Authors:** Mengzhu Wang, Mehdi Toghyani, Shemil P. Macelline, Andreas Lemme, Andrew J. Holmes, Peter H. Selle, Sonia Y. Liu

**Affiliations:** 1 School of Life and Environmental Sciences, Faculty of Science, The University of Sydney, Camperdown, 2006 NSW Australia; 2 Poultry Research Foundation, The University of Sydney, Camden, 2570 NSW Australia; 3 Evonik Operations GmbH, 63457 Hanau-Wolfgang, Germany; 4 Charles Perkins Centre, The University of Sydney, Camperdown, 2006 NSW Australia; 5 Sydney School of Veterinary Science, Camden, 2570 NSW Australia

**Keywords:** Amino acids, Broiler chickens, Crude protein, Sorghum, Wheat

## Abstract

**Background:**

Wheat and, to a lesser extent, sorghum are the dominant feed grains in Australian chicken-meat production. There is considerable local interest in the development of reduced-crude protein (CP) broiler diets in part because this would decrease the need to import soybean meal into the country. Maize is rarely included in Australian broiler diets, but birds appear better able to accommodate dietary CP reductions with maize than with wheat-based diets. Sorghum is more similar to maize than wheat and for this reason wheat- and sorghum-based diets, with standard and reduced-CP concentrations, were evaluated in broiler chickens in a direct comparison.

**Results:**

Reducing dietary CP from 205 to 175 g/kg CP did not statistically influence weight gain and FCR in broilers offered sorghum-based diets from 14 to 35 d post-hatch. In contrast, the 30 g/kg CP reduction compromised weight gain by 10.1% (1,964 versus 2,187 g/bird) and FCR by 9.68% (1.575 versus 1.436), in broilers offered wheat-based diets. Consequently, treatment interactions (*P* < 0.001) were observed for dietary CP levels grain type for both weight gain and FCR. Another treatment interaction (*P* < 0.001) was observed for starch digestibility coefficients in the distal jejunum. Birds offered 205 g/kg CP, wheat-based diets had superior starch digestibility by 11.6% (0.914 versus 0.819), but sorghum supported superior starch digestibility by 9.70% (0.837 versus 0.763) in the context of 175 g/kg CP diets.

**Conclusions:**

Under the condition of thid study, broiler chickens offered sorghum-based diets had a greater capacity to accommodate dietary CP reductions than their counterparts offered wheat-based diets. This study confirmed that wheat-based diets are not conducive to CP reductions, but the causal factors have yet to be identified precisely.

## Introduction

There are several advantages stemming from crude protein (CP) reductions in diets for broiler chickens including decreased nitrogen (N) excretion and ammonia (NH_3_) emissions coupled with improved litter quality resulting in enhanced bird welfare [1, 2]. Moreover, soybean meal inclusions are considerably lower in reduced-CP diets. Australia, and many other countries, imports substantial quantities of soybean meal from South America, which is not compatible with sustainable chicken-meat production [3]. Thus, the adoption of reduced-CP diets would be beneficial for the chicken-meat industry but is thwarted by observations that wheat-based diets are less conducive to CP reductions than maize, which was clearly demonstrated by Chrystal et al. [4]. Wheat and sorghum are the commonly used feed grains in Australian broiler diets and wheat is dominant, but maize is rarely used. As discussed by Selle et al. [5, 6], there is almost certainly numerous possible factors that may be contributing to the inferiority of wheat in the context of reduced-CP diets.

However, in comparison to wheat, sorghum and maize are relatively similar in several possibly important respects. Starch digestion rates in maize and sorghum are slower than wheat under both in vitro [7] and in vivo [8] conditions. Slowly digestible starch sources have been shown to advantage broiler performance [9], and it has been suggested that this may be related to more sustained pancreatic insulin secretions [10]. The amino acid profiles of maize and sorghum are quite similar and both feed grains usually have lower protein (N) contents than wheat. In one Australian survey [11], the average crude protein content of 27 wheat samples was 115.5 g/kg (range: 88 to 162), 17 sorghum samples was 101.9 g/kg (range: 71 to 118) and 7 maize samples was 80.0 g/kg (range: 76 to 87). As a result, wheat-based diets contain more non-bound amino acids (NBAA) to meet targeted amino acid specifications than reduced-CP diets based on maize or sorghum [12] and there is probably a ceiling on NBAA inclusions, which, if exceeded, compromises growth performance [13]. This ceiling is likely imposed by post-enteral imbalances between protein-bound and NBAA and catabolism of surplus amino acids [14]. Also, maize and sorghum are ‘non-viscous’ grains; whereas, wheat contains higher concentrations of soluble non-starch polysaccharides (NSP), which increases gut viscosity [15]. However, the routine inclusions of NSP-degrading enzymes in wheat-based diets should be remedial.

Sorghum is often considered to be inferior to maize and wheat as a feedstuff for broiler chickens which appears to stem from a ‘Bermuda triangle’ of anti-nutritive factors including kafirin, phenolic compounds and phytate [16]. However, Australian sorghum crops do not presently contain condensed tannin, the toxic hallmark of ‘bird-proof’ sorghums [17], which is a distinct benefit. However, sorghum- and wheat-based diets for broiler chickens with CP contents of 170 g/kg and 104 arginine to lysine ratios were directly compared by Macelline et al. [18]. The sorghum-based diet supported significant advantages in weight gain of 6.08% (2,147 versus 2,024 g/bird) and FCR of 4.89% (1.437 versus 1.511) and a numerical advantage in feed intake of 0.82% (3,083 versus 3,058 g/bird) from 14 to 35 d post-hatch. This outcome suggests that sorghum is better than wheat in the context of reduced-CP diets. Therefore, the objective of the present experiment was to confirm this outcome with the provision of diets based on sorghum or wheat with CP contents of 205 or 175 g/kg to straight-run broiler chickens in a 2 × 2 factorial array of dietary treatments.

## Materials and methods

The feeding study complied with specific guidelines approved by the Research Integrity and Ethics Administration of The University of Sydney (Project No. 2023/2316).

### Experiment design

The experimental design consisted of a 2 × 2 factorial array of treatments with two levels of dietary CP (205 versus 175 g/kg) and two feed grains (wheat or sorghum), which were offered to a total of 288 Ross 308 straight-run broiler chickens from 14 to 35 d post-hatch.

### Diet preparation

The experimental diets were formulated in accordance with the breeder’s recommendations and were based on near-infrared spectroscopy (NIR) of wheat, sorghum and soybean meal by the AMINONir Advanced program (Evonik Operations GmbH, Hanau, Germany). The analysed CP concentrations of wheat and sorghum were 123 and 104 g/kg, respectively, and the NIR predicted energy densities were 13.22 and 13.67 MJ/kg ME, respectively. The experimental diets were formulated to contain 12.9 MJ/kg metabolizable energy, 11.2 g/kg standardized ileal digestible (SID) lysine, 8.86 g/kg of SID digestible sulfur-containing amino acids, 7.49 g/kg of SID digestible threonine, 8.52 g/kg SID digestible valine and 7.71 g/kg SID digestible isoleucine. In addition, all four diets contained minimal levels of 12.3 g/kg SID digestible leucine, 12.1 g/kg SID digestible arginine, 4.21 g/kg SID digestible histidine, 1.79 g/kg SID digestible tryptophan, 14.5 g/kg glycine equivalent based on the recommendations by Wu [19]. The dietary composition and nutrient specifications of the experimental diets are listed in Tables 1 and 2. NBAA inclusions in wheat-based diets were increased from 10.1 to 47.4 g/kg with the dietary CP reduction; similarly, the increase in sorghum-based diets was from 8.8 to 45.9 g/kg.

**Table 1:** Compositi﻿on of experimental diets

Feed ingredient, g/kg	Wheat 205 g/kg CP	Wheat 175 g/kg CP	Sorghum 205 g/kg CP	Sorghum 175 g/kg CP
Wheat 12%	647	822	0.00	0.00
Sorghum 10%	0.00	0.00	623	745
Soybean meal 46.0%	259	66	282	131
Canola oil	40.2	10.2	39.4	21.3
DL-Methionine	3.40	4.11	3.45	3.92
Glycine	0.00	5.08	0.00	4.88
L-Arginine	1.11	6.34	1.03	5.31
L-Histidine	0.00	1.67	0.00	1.47
L-Isoleucine	0.40	3.43	0.00	2.43
L-Leucine	0.00	4.60	0.00	1.04
L-Lysine-HCl	2.87	7.30	2.72	6.31
L-Phenylalanine	0.00	3.06	0.00	2.28
L-Threonine	1.71	4.19	1.44	3.44
L-Tryptophane	0.00	0.43	0.00	0.44
L-Valine	0.61	3.63	0.16	2.59
L-Cysteine	0.00	0.81	0.00	0.83
L-Glutamine	0.00	1.00	0.00	8.83
L-Proline	0.00	1.73	0.00	2.11
Limestone	11.7	12.1	11.4	11.0
MDP^a^	4.19	6.23	5.26	9.21
Potassium carbonate	0.00	4.70	0.00	3.92
Sodium bicarbonate	2.82	5.80	3.51	6.06
Vitamin-mineral premix^b^	2.00	2.00	2.00	2.00
Choline chloride 75%	0.62	1.18	1.24	1.83
Xylanase^c^	0.20	0.20	0.20	0.20
Phytase^d^	0.10	0.10	0.10	0.10
Celite	20.0	20.0	20.0	20.0
Salt	2.01	0.00	1.68	0.00
Total non-bound amino acids	9.47	45.77	8.20	44.49

^a^MDP Mono-dicalcium phosphate

^b^Vitamin-trace mineral premix supplies in IU/kg or mg/kg of diet: retinol, 12,000 IU; cholecalciferol, 5,000 IU; tocopheryl acetate, 75 mg; menadione, 3 mg; thiamine, 3 mg; riboflavin, 8 mg; niacin, 55 mg; pantothenate, 13 mg; pyridoxine, 5 mg; folate, 2 mg; cyanocobalamin, 16 μg; biotin, 200 μg; cereal-based carrier, 149 mg; Cu (sulphate), 16 mg; Fe (sulphate), 40 mg; I (iodide), 1.25 mg; Se (selenate), 0.3 mg; Mn (sulphate and oxide),120 mg; Zn (sulphate and oxide), 100 mg; cereal-based carrier, 128 mg

^c^Xylanase 40,000 U/g (Danisco, DuPont Nutrition & Bioscience)

^d^Phytase 10,000 FTU/kg (Axtra PHY, Danisco, DuPont Nutrition & Bioscience)

**Table 2:** Nutrient specifications of experimental diets

Nutrient, g/kg	Wheat 205 g/kg CP	Wheat 175 g/kg CP	Sorghum 205 g/kg CP	Sorghum 175 g/kg CP
Dry matter	912	912	900	899
Metabolisable energy, MJ/kg	12.9	12.9	12.9	12.9
Crude Protein	205	175	205	175
Starch	410	518	395	471
Glutamic acid	41.9	35.0	32.1	32.0
Lysine^a^	11.2	11.2	11.2	11.2
Methionine	5.84	5.74	6.17	6.02
Total sulfur amino acids	8.86	8.86	8.86	8.86
Threonine	7.49	7.49	7.49	7.49
Valine	8.52	8.52	8.52	8.52
Isoleucine	7.71	7.71	7.71	7.71
Leucine	12.6	12.3	14.9	12.3
Arginine	12.1	12.1	12.1	12.1
Histidine	4.28	4.28	4.21	4.28
Tryptophan	2.26	1.79	2.13	1.79
Tyrosine	6.57	5.20	8.31	5.76
Glycine equivalents^b^	14.9	14.5	14.5	14.5
Phenylalanine	8.48	8.40	8.69	8.40
Calcium	8.00	8.00	8.00	8.00
Available P	4.00	4.00	4.00	4.40
Total P	5.03	4.80	4.34	4.40
Crude fibre	25.0	21.5	24.7	21.5
Crude fat	54.7	26.4	59.9	44.0
Sodium	1.90	1.90	1.90	1.90
Potassium	7.32	7.18	7.47	7.40
Chloride	2.40	2.34	2.40	2.37
DEB, mEq/kg^c^	202	200	206	205

^a^All amino acids are expressed on standardized ileal digestible basis

^b^Glycine equivalents = glycine concentration + (serine concentration × 0.7143)

^c^DEB: dietary electrolyte balance mEq/kg = K^+^ + Na^+^ −Cl^−^

Wheat typically contains more glutamic acid, or glutamine plus glutamate, than sorghum. In one local survey [11], 27 wheat samples had an average glutamic acid concentration of 36.7 g/kg, as opposed 20.9 g/kg in 17 sorghum samples. Thus, wheat contained 75.6% more glutamic acid than sorghum. To maintain similar concentrations of glutamic acid and non-essential amino acids in reduced 175 g/kg CP diets, glutamine was added at 8.83 g/kg to the reduced-CP sorghum-based diet and at 1.00 g/kg in the wheat-based diet. This adjustment brought analysed glutamic acid concentrations in the 175 g/kg CP diets to parity with 33.4 and 33.9 g/kg glutamic acid in the wheat and sorghum-based diets, respectively.

Wheat and sorghum were ground through 4.0 mm hammer-mill screen prior to incorporation with the other ingredients, then the diets were steam-pelleted through a Palmer PP330 pellet press (Palmer Milling Engineering, Griffith, NSW, Australia) at a conditioning temperature of 80 °C with a residence time of 14 s. Subsequently, the diets were cooled in a vertical cooler to ambient temperature. All experimental diets were supplemented with xylanase and phytase and an acid insoluble ash marker (Celite™ World Minerals, Lompoc, CA, USA) was included at 20 g/kg in all diets to determine digestibility coefficients. The analysed nutrient composition of the experimental diets is shown in Table 3. The analysed content of the reduced-CP, sorghum-based diet was higher (180 versus 167 g/g) than that of the corresponding wheat-based diet. This apparent aberration is largely due to the relatively high 192 g/kg N content of glutamine.

**Table 3:** Analysed nutrient concentrations in experimental diets

Item, g/kg	Wheat 205 g/kg CP	Wheat 175 g/kg CP	Sorghum 205 g/kg CP	Sorghum 175 g/kg CP
Dry matter	88.1	86.9	87.6	87.9
Crude protein	198	167	205	180
Digestible crude protein^a^	179	151	177	149
Starch	384	480	13	469
Arginine	11.6	10.9	11.5	11.5
Histidine	4.7	4.2	4.9	4.4
Isoleucine	8.1	7.2	8.6	7.7
Leucine	13.5	12.5	18.6	15.5
Lysine	11.2	10.6	11.5	10.9
Methionine	4.6	4.4	4.6	4.6
Phenylalanine	8.9	7.5	9.7	6.8
Threonine	8.0	6.9	8.3	7.6
Valine	9.1	8.4	9.6	8.7
Alanine	7.2	4.6	10.8	8.9
Aspartic acid	15.9	8.3	18.1	11.3
Glutamic acid	41.4	33.4	36.3	33.9
Glycine	7.8	9.0	7.3	8.4
Proline	12.8	12.5	11.7	13.1
Serine	9.1	6.0	9.1	6.2
Tyrosine	4.2	2.6	4.3	3.0

^a^Digestible crude protein on dry matter basis = dietary crude protein × apparent ileal digestibility coefficient of crude protein

### Bird management

A total of 288 mixed sex Ross 308 chicks were procured from a commercial hatchery and offered a common starter with 12.5 MJ/kg ME and 12.8 g/kg digestible lysine from 0 to 13 d post-hatch. At 14 d post-hatch, birds were individually identified (wing-tags), weighed and distributed amongst 48 bioassay cages (750 mm in width and length, 510 mm in height) so that cage body weight means and variations within cages were statistically similar. The mean body weight of the 48 cages was 548 ± 4.5 g/bird at 14 d post-hatch. Each of four dietary treatments were offered to 12 replicate cages (six birds per cage) to 35 d post-hatch. Birds had unrestricted access to feed and water in an environmentally controlled facility with 23-h-on-1-h-off lighting regime for the first 3 d and 18-h-on-6-h-off lighting regime during the rest of the study. An initial room temperature of 32 ± 1 °C was maintained for the first week, which was gradually decreased to 22 ± 1 °C by the end of the third week and maintained at this temperature for the duration of the feeding study.

### Data and sample collection, chemical analyses, calculations

Growth performance (weight gain, feed intake, FCR) of each bird was determined from 14 to 35 d post-hatch. The bodyweight and gender of any dead or culled birds were recorded daily to correct feed intakes and adjust FCR calculations for relevant cages. Total excreta outputs and feed intakes were recorded from 28 to 30 d post-hatch to determine parameters of nutrient utilization by the classic approach. These parameters included apparent metabolizable energy (AME), metabolizable to gross energy ratios (ME:GE ratios), nitrogen (N) retention and N-corrected AME (AMEn). Excreta was weighed before and after it was dried in a forced-air oven at 80 °C for 24 h to determine excreta dry matter. The gross energy (GE) of excreta and diets were determined using an adiabatic bomb calorimeter (Parr Instrument Company, Moline,. Illinois, USA). The AME values of the dies on a dry matter basis were calculated from the following equation:

$$\text{AME}=\frac{(\text{GE diet }\times \text{Feed intake})-\left(\text{GE excreta }\times \text{Excreta weight}\right)}{(\text{Feed intake})}$$

ME:GE ratios were calculated by dividing AME by the gross energy (GE) of the appropriate diets. N contents of diets and excreta were determined using a nitrogen determinator (Leco Corporation, St Joseph, MI, USA) and N retentions calculated from the following equation:

$$\text{N retention}=\frac{(\text{N diet }\times \text{Feed intake})-\left(\text{N excreta }\times \text{Excreta weight}\right)}{(\text{N diet }\times \text{Feed intake})}\times 100$$

N-corrected AME (AMEn, MJ/kg DM) values were calculated by correcting N retention to zero using the factor of 36.54 kJ/g N retained in the body [20].

At d 35, all the birds from each cage were individually weighed and euthanised by intravenous injections of sodium pentobarbitone, the abdominal cavity opened, small intestines removed and gender was determined. Digesta samples were collected in their entirety from the distal jejunum and distal ileum. The distal jejunum was demarcated by the mid-point between the end of the duodenal loop and Meckel’s diverticulum and the distal ileum was demarcated by the mid-point between Meckel’s diverticulum proximally and the ileo-caecal junction distally. Digesta were manually expressed gently from segments and samples pooled for each pen, homogenized, freeze-dried, and ground through 0.5 mm screen to analyse for starch, protein (N) and amino acids concentrations. In addition, abdominal fat-pads were dissected out, weighed and recorded against final body-weights to calculate relative abdominal fat-pad weights. Also, Pectoralis major, Pectoralis minor, and leg quarters were removed from the carcass and recorded against final body-weights to calculate relative weights of carcass traits.

Starch concentrations in feed and digesta samples were determined by using total starch assay kits (Megazyme, Wicklow, Ireland) as described by Mahasukhonthachat et al. [21]. Nitrogen contents of diets and digesta were determined using a nitrogen determinator (Leco Corporation, St Joseph, MI, USA) by the Dumas method and AIA concentrations were determined by the method described by Siriwan et al. [22]. Amino acid concentrations of diets and digesta were determined by 24-h liquid hydrolysis at 110 °C in 6 mol/L HCl followed by analysis of 16 amino acids using the Waters AccQTag Ultra chemistry on a Waters Acquity UPLC (Waters Corporation, Milford, Massachusetts, USA). The apparent digestibility coefficients for starch, protein (N) and amino acids sites were calculated from the following equation:

$$\text{Digestibilit coefficient }=\frac{\left(\text{Nutrient diet}/\text{AIA diet}\right) -(\text{Nutriten digesta}/\text{AIA digesta})}{\text{Nutrient diet}/\text{AIA diet}}$$

Disappearance rates (g/bird/d) of starch and protein (N) were calculated from the following equation:

$$\text{Nutrient disappearance rate }=\text{Feed intake }\times \text{dietary nutrient }\times \text{digestibility coefficient}$$

Ratios of starch to protein disappearance rates in the distal ileum were calculated as this eliminates the confounding influence of feed intake. Blood samples (brachial vein) were taken at 105 min post-prandially to determine plasma concentrations of 20 free proteinogenic amino acids by standard analytical procedures.

### Statistical analysis

The experimental data were analysed by two-way analyses of covariance (ANCOVA) using the JMP Pro 16.0 software package (JMP Statistical Discovery LLC, Cary, NC, USA). The ratio of male bird numbers to total bird numbers in each cage was used as the covariant. Linear and quadratic relationships and multiple linear regressions were established when considered appropriate. Cage means were the experimental unit and a probability level of less than 5% was considered statistically significant by Tukey HSD test.

## Results

### Growth performance

The effect of dietary treatments on growth performance and carcass traits are shown in Table 4. Treatment interactions (*P* < 0.001) were observed for weight gain and FCR. The reduction in dietary CP significantly reduced weight gain in broiler chickens offered wheat-based diets by 10.1% (1,964 versus 2,184 g/bird); whereas, the CP reduction did not influence weight gain of birds offered sorghum-based diets. The same dietary CP reduction significantly elevated FCR in birds offered wheat-based diets by 9.68% (1.575 versus 1.436), but sorghum-based diets did not influence feed conversion efficiency. Feed intake was not influenced by dietary treatment and the acceptable overall mortality rate of 2.27% was not influenced by treatment. A treatment interaction (*P* < 0.001) was observed for relative abdominal fat-pad weights. Broilers offered sorghum-based diets had 25.5% heavier fat-pad weights by 25.5% (10.89 versus 8.68 g/kg) compared to their wheat-based counterparts on 175 g/kg CP diets, but there was not a difference between feed grains in birds offered 205 g/kg CP diets.

**Table 4:** The effects of dietary treatments on growth performance and carcass traits from 14 to 35 d post-hatch

Treatment		Growth performance				Carcass trait, g/kg			
CP	Grain	Weight Gain, g/bird	Feed intake, g/bird	FCR, g/g	Mortality, %	Fad-pad	Pectoralis major	Pectoralis minor	Leg quarters
205	Wheat	2,184^a^	3,135	1.436^c^	1.93	8.77^b^	206^ab^	22.6	196
	Sorghum	2,122^b^	3,076	1.450^bc^	2.44	8.01^b^	212^a^	19.8	198
175	Wheat	1,964^c^	3,095	1.575^a^	1.05	8.68^b^	211^a^	20.0	198
	Sorghum	2,096^b^	3,067	1.464^b^	3.65	10.89^a^	197^b^	18.5	202
SEM		18.9	24.2	0.0089	0.012	0.364	3.4	1.58	3.9
Main effects: CP									
205		2,153	3,106	1.443	2.19	8.39	209	21.2	197
175		2,030	3,081	1.520	2.35	9.79	204	19.2	200
Main effects: Grain									
Wheat		2,074	3,115	1.506	1.49	8.73	209	21.3	197
Sorghum		2,109	3,072	1.457	3.05	9.45	204	19.1	200
Significance (*P*)									
Crude protein (CP)		< 0.001	0.321	< 0.001	0.887	< 0.001	0.204	0.165	0.458
Feed grain (FG)		0.036	0.075	< 0.001	0.173	0.047	0.241	0.274	0.508
CP × FG interaction		< 0.001	0.522	< 0.001	0.372	< 0.001	0.010	0.702	0.894

Means within columns not sharing a similar superscript are significantly different at the 5% level of probability

*SEM *Standard error of mean

### Carcass traits

A treatment interaction (*P* = 0.010) was observed for Pectoralis major yields. They were similar with 205 g/kg CP diets, but significantly depressed by 7.08% (197 versus 212 g/kg) in birds offered sorghum-based diets following the dietary CP reductions as opposed to their wheat-based counterparts. Dietary treatment did not influence relative weights of Pectoralis minor and leg quarters.

### Nutrient utilisation

There were not any significant effects of dietary treatment on energy utilisation (AME, AME:GE, AMEn) as displayed in Table 5. However, dietary CP reductions enhanced N-retention by 5.6 percentage units (69.7% versus 64.1%; *P* < 0.001) as a main effect.

**Table 5:** The effects of dietary treatments on nutrient utilisation from 28 to 30 d post-hatch

Treatment		Nutrient utilisation			
CP	Grain	AME, MJ/kg DM	AME:GE, MJ/MJ	N retention, %	AMEn, MJ/kg DM
205	Wheat	12.75	0.788	64.4	11.34
	Sorghum	12.52	0.773	63.8	11.22
175	Wheat	12.78	0.787	68.3	11.36
	Sorghum	12.66	0.778	71.0	11.20
SEM		0.185	0.0134	1.39	0.189
Main effects: CP					
205		12.63	0.781	64.1^b^	11.28
175		12.72	0.782	69.7^a^	11.28
Main effects: Grain					
Wheat		12.76	0.788	66.4	11.35
Sorghum		12.59	0.775	67.4	11.21
Significance (*P*)					
Crude protein (CP)		0.667	0.892	< 0.001	0.991
Feed grain (FG)		0.352	0.357	0.464	0.464
CP × FG interaction		0.790	0.837	0.243	0.943

Means within columns not sharing a similar superscript are significantly different at the 5% level of probability

*SEM *Standard error of mean

### Starch and protein (N) digestibility coefficients

The effects of dietary treatments on the apparent jejunal and ileal digestibility coefficients and disappearance rates of starch and protein (N) and disappearance rate ratios are shown in Table 6. Significant treatment interactions were observed for starch digestibility coefficients in jejunum (*P* < 0.001) and ileum (*P* = 0.031). In the jejunum, wheat in 205 g/kg CP diets supported superior starch digestibility by 11.6% (0.914 versus 0.819) but sorghum supported higher starch digestibility by 9.70% (0.837 versus 0.763) following the CP reduction to 175 g/kg. Wheat-based diets supported higher protein (N) digestibility coefficients in the jejunum by 17.3% (0.690 versus 0.588; *P* < 0.001) and ileum by 6.74% (0.792 versus 0.742; *P* = 0.010). A treatment interaction (*P* = 0.005) was observed for the jejunal starch disappearance rate as wheat supported the more rapid rate in 205 g/kg CP diets by 5.51% (52.27 versus 49.54 g/bird/d), but sorghum supported the more rapid rate by 3.75% (57.31 versus 55.24 g/bird/d) in 175 g/kg CP diets. Lower CP diets supported the faster starch disappearance rate by 16.0% (62.79 versus 54.12 g/bird/d) in the distal ileum. Protein (N) disappearance rates were more rapid in the jejunum by 9.73% (18.38 versus 16.75 g/bird/d; *P* = 0.015) and ileum by 19.8% (23.15 versus 19.33 g/bird/d) in birds offered 205 g/kg CP diets. A treatment interaction (*P* = 0.007) was detected for jejunal starch:protein disappearance rate ratios because the ratio was significantly higher in birds offered 175 g/kg sorghum-based diets (3.75 versus 3.02), but not 205 g/kg CP diets. Ileal starch:protein disappearance rate ratios were higher (3.33 versus 2.35; *P* < 0.001) in birds offered 175 g/kg CP diets.

**Table 6:** The effects of dietary treatments on apparent digestibility coefficients and disappearance rates of starch and protein (N) in distal jejunum (DJ) and distal ileum (DI) and starch:protein disappearance rate ratios at 35 d post-hatch

Treatment		Digestibility of starch		Digestibility of protein N		Disappearance rates of starch, g/bird/d		Disappearance rates of protein, g/bird/d		Disappearance rate ratio of starch:protein	
CP	Grain	DJ	DI	DJ	DI	DJ	DI	DJ	DI	DJ	DI
205	Wheat	0.914^a^	0.958^a^	0.653	0.796	52.27^bc^	53.94	19.30	23.54	2.74^b^	2.30
	Sorghum	0.819^b^	0.916^b^	0.582	0.756	49.54^c^	54.29	17.47	22.76	2.87^b^	2.41
175	Wheat	0.763^c^	0.915^b^	0.727	0.787	55.24^ab^	63.82	17.87	19.58	3.02^b^	3.28
	Sorghum	0.837^b^	0.922^ab^	0.594	0.728	57.31^a^	61.76	15.63	19.08	3.75^a^	3.32
SEM		0.0135	0.0107	0.0223	0.0782	0.800	0.764	0.637	0.548	0.110	0.100
Main effects: CP											
205		0.866	0.937	0.617	0.776	50.90	54.12^b^	18.38^a^	23.15^a^	2.80	2.35^b^
175		0.800	0.918	0.660	0.758	56.28	62.79^a^	16.75^b^	19.33^b^	3.39	3.33^a^
Main effects: Grain											
Wheat		0.839	0.937	0.690^a^	0.792^a^	57.75	58.88	18.59^a^	21.56	2.88	2.79
Sorghum		0.828	0.919	0.588^b^	0.742^b^	53.42	58.02	16.55^b^	20.92	3.31	2.96
Significance (*P*)											
Crude protein (CP)		< 0.001	0.088	0.065	0.340	< 0.001	< 0.001	0.015	< 0.001	< 0.001	< 0.001
Feed grain (FG)		0.424	0.101	< 0.001	0.010	0.680	0.256	0.002	0.246	< 0.001	0.370
CP × FG interaction		< 0.001	0.031	0.181	0.616	0.005	0.137	0.750	0.804	0.007	0.642

Means within columns not sharing a similar superscript are significantly different at the 5% level of probability

*SEM* Standard error of mean

### Apparent amino acid digestibility coefficients

The effects of dietary treatments on apparent jejunal and ileal digestibility coefficients of amino acids are shown in Tables 7 and 8, respectively. In the distal jejunum, significant treatment interactions were observed for isoleucine, leucine, phenylalanine, valine, serine and tyrosine. The genesis of these interactions was the decreases in amino acid digestibilities in birds offered 175 g/kg, sorghum-based diets. As main effects, the dietary CP reduction significantly increased jejunal digestibilities of total amino acids (7.56%) plus arginine (8.85%), histidine (9.21%), lysine (10.0%), threonine (10.1%), glutamic acid (5.95%), glycine (23.2%) and proline (11.1%), as shown in parentheses. Wheat-based diets supported significant increases in digestibilities of histidine (12.0%), methionine (5.24%), threonine (10.8%), glycine (9.35%), proline (21.9%) and total amino acids (14.7%).

**Table 7:** The effects of dietary treatments on apparent digestibility coefficients amino acids in distal jejunum at 35 d post-hatch

Treatment		Amino acids																
CP	Grain	Arg	His	Ile	Leu	Lys	Met	Phe	Thr	Val	Ala	Asp	Glu	Gly	Pro	Ser	Tyr	Total
205	Wheat	0.756	0.706	0.687^b^	0.678^b^	0.719	0.867	0.709^b^	0.669	0.674^b^	0.629	0.639	0.798	0.640	0.775	0.673^ab^	0.656^a^	0.720
	Sorghum	0.758	0.661	0.635^b^	0.622^bc^	0.735	0.842	0.653^b^	0.632	0.621^b^	0.609	0.638	0.683	0.600	0.633	0.616^b^	0.562^b^	0.657
175	Wheat	0.848	0.806	0.808^a^	0.809^a^	0.828	0.898	0.810^a^	0.768	0.796^a^	0.641	0.642	0.856	0.806	0.858	0.710^ab^	0.701^a^	0.807
	Sorghum	0.801	0.688	0.662^b^	0.602^c^	0.772	0.837	0.588^c^	0.666	0.652^b^	0.574	0.564	0.712	0.722	0.707	0.534^c^	0.469^c^	0.673
SEM		0.0157	0.0179	0.0201	0.0214	0.0192	0.0125	0.0210	0.0798	0.0203	0.0238	0.0959	0.0631	0.0192	0.0153	0.0237	0.0282	0.0187
Main effects: CP																		
205		0.757^b^	0.684^b^	0.661	0.650	0.727^b^	0.854	0.681	0.651^b^	0.648	0.619	0.638	0.740^b^	0.620^b^	0.704^b^	0.644	0.609	0.688^b^
175		0.824^a^	0.747^a^	0.735	0.706	0.800^a^	0.867	0.699	0.717^a^	0.724	0.608	0.603	0.784^a^	0.764^a^	0.782^a^	0.622	0.585	0.740^a^
Main effects: Grain																		
Wheat		0.802	0.756^a^	0.747	0.744	0.773	0.883^a^	0.759	0.719^a^	0.735	0.635	0.641	0.827	0.723^a^	0.817^a^	0.692	0.679	0.763^a^
Sorghum		0.779	0.675^b^	0.649	0.612	0.754	0.839^b^	0.620	0.649^b^	0.637	0.591	0.601	0.697	0.661^b^	0.670^b^	0.575	0.515	0.665^b^
Significance (*P*)																		
Crude protein (CP)		0.000	0.001	0.001	0.014	0.001	0.307	0.388	0.002	0.001	0.635	0.154	0.010	0.001	0.001	0.364	0.415	0.010
Feed grain (FG)		0.142	0.001	0.001	0.001	0.303	0.001	0.001	0.001	0.001	0.068	0.096	0.001	0.002	0.001	0.001	0.001	0.001
CP × FG interaction		0.135	0.053	0.028	0.001	0.072	0.163	0.000	0.119	0.035	0.333	0.122	0.381	0.256	0.768	0.018	0.021	0.064

Means within columns not sharing a similar superscript are significantly different at the 5% level of probability

*SEM *Standard error of mean

**Table 8:** The effects of dietary treatments on apparent digestibility coefficients amino acids in distal ileum at 35 d post-hatch

Treatment		Amino acids																
CP	Grain	Arg	His	Ile	Leu	Lys	Met	Phe	Thr	Val	Ala	Asp.	Glu	Gly	Pro	Ser	Tyr	Total
205	Wheat	0.894	0.852	0.851	0.848	0.865	0.949	0.865^a^	0.818	0.837	0.802	0.808	0.901	0.808	0.883	0.837	0.844	0.861
	Sorghum	0.893	0.816	0.819	0.814	0.863	0.934	0.834^a^	0.787	0.803	0.791	0.806	0.841	0.782	0.799	0.806	0.790	0.824
175	Wheat	0.904	0.868	0.868	0.873	0.874	0.942	0.879^a^	0.818	0.856	0.714	0.739	0.908	0.866	0.907	0.802	0.802	0.868
	Sorghum	0.896	0.799	0.808	0.772	0.870	0.918	0.774^b^	0.784	0.794	0.730	0.734	0.830	0.826	0.809	0.732	0.700	0.806
SEM		0.0106	0.0140	0.0164	0.0180	0.0142	0.9358	0.0169	0.0174	0.0169	0.0226	0.0184	0.0137	0.0149	0.0136	0.0185	0.0221	0.0154
Main effects: CP																		
205		0.894	0.834	0.835	0.831	0.864	0.942	0.849	0.802	0.820	0.796^a^	0.807^a^	0.871	0.795	0.841	0.822^a^	0.817^a^	0.843
175		0.900	0.834	0.838	0.823	0.872	0.930	0.826	0.801	0.825	0.722^b^	0.737^b^	0.869	0.846	0.858	0.767^b^	0.751^b^	0.837
Main effects: Grain																		
Wheat		0.899	0.860^a^	0.860^a^	0.861^a^	0.870	0.946^a^	0.872	0.818	0.846^a^	0.758	0.774	0.905^a^	0.837	0.895^a^	0.820^a^	0.823^a^	0.864^a^
Sorghum		0.895	0.808^b^	0.813^b^	0.793^b^	0.867	0.926^b^	0.804	0.785	0.798^b^	0.760	0.770	0.836^b^	0.804	0.804^b^	0.769^b^	0.745^b^	0.815^b^
Significance (*P*)																		
Crude protein (CP)		0.540	0.964	0.861	0.635	0.568	0.194	0.181	0.948	0.779	0.002	0.001	0.867	0.002	0.228	0.006	0.005	0.704
Feed grain (FG)		0.658	0.000	0.006	0.000	0.838	0.028	0.000	0.063	0.006	0.898	0.850	0.001	0.028	0.001	0.008	0.001	0.002
CP × FG interaction		0.744	0.258	0.398	0.074	0.913	0.602	0.037	0.922	0.420	0.550	0.951	0.515	0.663	0.641	0.309	0.287	0.424

Means within columns not sharing a similar superscript are significantly different at the 5% level of probability

*SEM *Standard error of mean

One treatment interaction (*P* = 0.037) was observed in the distal ileum which was for phenylalanine because wheat supported significantly higher digestibility than sorghum by 13.6% (0.879 versus 0.774) following the transition to 175 g/kg CP diets. As main effects, the CP reduction diets supported significantly higher digestibilities of alanine (10.2%), aspartic acid (9.50%), serine (7.17%) and tyrosine (8.79%) than sorghum-based diets. Again, as main effects, wheat-based diets generated significantly higher digestibilities of histidine (6.44%), isoleucine (5.78%), leucine (8.58%), valine (6.02%), glutamic acid (8.25%), proline (11.3%), serine (6.63%), tyrosine (10.5%) and total amino acids (6.01%) than sorghum-based diets.

### Amino acid plasma concentrations

Plasma concentrations of free amino acids in birds fasted for 105 min are shown in Tables 9 and 10. Treatment interactions were observed for plasma levels of leucine (*P* = 0.034) and methionine (*P* = 0.049) across the essential amino acids. Sorghum-based diets supported significantly higher leucine levels than wheat-based diets by 20.5% (24.7 versus 20.5 µg/mL) with 205 g/kg CP diets but numerically lower leucine levels by 10.7% (15.9 versus 17.8 µg/mL) with the reduction to 175 g/kg CP. Methionine followed the same interactive pattern but the variations were less pronounced. As main effects, the dietary CP reduction significantly depressed concentrations of histidine (8.12%), isoleucine (23.8%) and phenylalanine (27.5%) as shown in parentheses. Sorghum-based diets supported significantly lower levels of isoleucine (19.3%) and valine (20.0%). The balance of essential amino acids was not statistically influenced by treatment. Across the non-essential amino acids, treatment interactions were observed for asparagine (*P* = 0.021) and serine (*P* = 0.016). Sorghum-based diets at 205 g/kg CP supported numerically lower asparagine levels by 29.8% (11.8 versus 16.8 µg/mL), but statistically higher levels by a 3.4-fold factor (10.4 versus 3.1 µg/mL) at 175 g/kg CP. Sorghum-based diets supported numerically higher serine levels by 23.0% (53.5 versus 43.5 µg/mL) at 205 g/kg CP, but numerically lower levels by 15.1% (50.0 versus 58.9 µg/mL) at 175 g/kg CP. As main effects, sorghum-based diets supported significantly higher alanine levels (21.7%), but lower levels of cysteine (19.9%) and proline (21.7%). The transition from 205 to 175 g/kg CP increased glutamine concentrations by 29.8% (208 versus 173 µg/mL; *P* = 0.006).

**Table 9:** Plasma concentrations of free essential amino acids (µg/mL) at 105 min post-prandial

Treatment		Arg	His	Ile	Leu	Lys	Met	Phe	Thr	Trp	Val
CP	Grain										
205	Wheat	82.4	7.2	13.5	20.5^b^	47.4	15.0	19.3	71.8	4.9	26.1
	Sorghum	76.2	6.1	10.9	24.7^a^	51.3	17.6	19.4	66.2	4.6	21.5
175	Wheat	77.7	5.6	10.3	17.8^b^	39.8	18.0	15.6	90.0	4.1	22.8
	Sorghum	75.1	4.0	8.4	15.9^b^	51.0	14.1	12.3	81.0	4.0	17.7
SEM		7.70	0.68	0.90	1.47	4.35	1.69	1.01	7.72	0.36	1.72
Main effect: CP											
205		79.3	6.6^a^	12.2^a^	22.6	49.3	16.3	19.3^a^	69.0	4.8	23.8
175		76.4	4.8^b^	9.3^b^	16.8	45.4	16.0	14.0^b^	85.5	4.0	20.3
Main effect: Feed grain											
Wheat		80.0	6.4	11.9^a^	19.1	43.6	16.5	17.4	80.9	4.5	24.5^a^
Sorghum		75.7	5.1	9.6^b^	20.3	51.1	15.8	15.8	73.6	4.3	19.6^b^
Significance (*P*)											
Crude protein (CP)		0.759	0.046	0.019	0.006	0.466	0.900	0.001	0.165	0.058	0.113
Feed grain (FG)		0.568	0.053	0.014	0.396	0.087	0.686	0.096	0.395	0.565	0.007
CP × FG interaction		0.796	0.742	0.699	0.034	0.359	0.049	0.069	0.832	0.774	0.907

Means within columns not sharing a similar superscript are significantly different at the 5% level of probability

*SEM *Standard error of mean

**Table 10:** Plasma concentrations of free non-essential amino acids (µg/mL) at 105 min post-prandial

Treatment		Ala	Asn	Asp	Cys	Gln	Glu	Gly	Pro	Ser	Tyr
CP	Grain										
205	Wheat	68.9	22.1	14.9	16.8^a^	166	26.8	55.3	56.2	43.5^b^	31.0
	Sorghum	79.1	27.4	12.5	11.8^a^	180	28.3	50.3	42.7	53.58^ab^	36.8
175	Wheat	52.8	19.1	13.3	3.1^b^	204	22.6	56.3	59.1	58.9^a^	22.9
	Sorghum	68.9	20.2	10.1	10.4^a^	212	24.1	53.0	47.7	50.0b^a^	20.7
SEM		6.82	4.06	1.04	2.43	11.3	2.53	4.60	5.88	4.41	2.41
Main effect: CP											
205		74.0	24.8	13.7	14.3	173^a^	27.6	53.0	49.4	48.5	33.9^a^
175		60.8	19.6	11.7	6.7	208^b^	23.3	54.7	53.4	54.5	21.8^b^
Main effect: Feed grain											
Wheat		60.8^b^	20.6	14.1^a^	9.9	185	24.7	55.8	57.7^a^	51.2	26.9
Sorghum		74.0^a^	23.8	11.3^b^	11.1	196	26.2	51.7	45.2^b^	51.8	28.7
Significance (*P*)											
Crude protein (CP)		0.184	0.404	0.100	0.006	0.006	0.197	0.690	0.567	0.349	0.002
Feed grain (FG)		0.035	0.327	0.327	0.642	0.327	0.550	0.383	0.049	0.883	0.387
CP × FG interaction		0.601	0.505	0.505	0.024	0.777	0.994	0.850	0.850	0.016	0.053

Means within columns not sharing a similar superscript are significantly different at the 5% level of probability

*SEM *Standard error of mean

## Discussion

Overall, across all treatments, Aviagen 2022 performance objectives for Ross 308 mixed-sex birds from 14 to 35 d post-hatch were surpassed by 18.7% (2,092 versus 1,763 g/bird) in weight gain, 15.3% (3,093 versus 2,682 g/bird) in feed intake and 2.63% (1.481 versus 1.521) in FCR. Birds offered 205 g/kg CP, wheat-based diets had significantly better weight gains by 2.92% with a numerical advantage of 0.97% in FCR in comparison to their sorghum-based counterparts. However, following the transition to reduced 175 g/kg CP diets, the sorghum-based diet significantly enhanced weight gain by 6.72% (2,096 versus 1,964 g/bird) and FCR by 7.05% (1.464 versus 1.575) in comparison to the corresponding wheat-based diet.

In the direct comparison [18], sorghum- and wheat-based diets with elevated arginine to lysine ratios of 110 in 170 g/kg CP diets were also evaluated. Sorghum-based diet supported significant advantages in weight gain of 9.20% (2,161 versus 1,979 g/bird) and FCR of 9.01% (1.404 versus 1.543). Thus, the growth performance advantages in favour of sorghum were amplified by the increase in arginine relative to lysine. These outcomes support the proposal that birds offered sorghum-based diets are better able to accommodate CP reductions than wheat-based diets; however, the underlying reasons for this difference require clarification.

One remarkable outcome in the present study was that the jejunal digestibility of starch in birds offered wheat-based diets declined by 16.5% (0.914 versus 0.763) pursuant to the CP reduction from 205 to 175 g/kg. Moreover, there were signicant negative correlations between starch jejunal digestibilities and 10 amino acids (arginine, histidine, isoleucine, leucine, lysine, phenylalanine, threonine, valine, glycine, and proline), from a total of 16, in wheat-based diets. In total contrast, all correlations in sorghum-based diets were positive with significant relationships for 9 amino acids, as shown in Table 11. The Moss et al. [23] study provides a precedent, but in reverse. In this study dietary CP levels were reduced by replacing maize grain with maize starch. The control diet had analysed starch concentration of 269 g/kg as opposed to an average of 436 g/kg in the five treatment diets containing an average 479 g/kg maize starch inclusion. Significant negative correlations were observed between apparent starch digestibility coefficients and 9 amino acids in the distal jejunum. As examples, distal jejunal digestibilities of arginine were depressed by up to 13.8% (0.656 versus 0.761) and phenylalanine by 16.7% (0.573 versus 0.688) in treatment diets versus the control. The implication is that in birds offered wheat-based diets, glucose and amino acids were competing for intestinal uptakes along the jejunum. Intestinal uptakes of glucose and amino acids are complex and interactive but there are indications that glucose and amino acids may compete for absorption along the small intestine [24]. That intestinal absorption capacity is rate-limiting in poultry was advanced by Croom et al. [25]. and glucose and amino acid intestinal uptakes are subject to mutual inhibition was Vinardell’s conclusion [26]. Following starch digestion by pancreatic α-amylase in the gut lumen, intestinal uptakes of glucose are predominantly via the apically located sodium glucose cotransporter SGLT-1 [27]. Similarly, intestinal uptakes of monomeric, or non-bound, amino acids are mainly conducted via several Na^+^-dependent transporters with overlapping amino acid afinities [28]. In contrast, the majority of protein-bound amino acids are absorbed as di- and tri-peptides via the oligopeptide transporter Pept-1, in conjunction with the sodium-hydrogen exchanger NHE, which is a more efficient system [29]. Thus, the transition from standard to reduced-CP diets, with increased NBAA inclusions and eleated starch contents, creates more scope for competition between glucose and monomeric amino acids for co-absorption with sodium and intestinal uptakes via their respective Na^+^-dependent transporters. Intestinal uptakes of these nutreints are driven by the sodium pump (Na^+^,K^+^-ATPase) in the baso-lateral membrane of enterocytes. The function of which is largely dependent on cystolic concentrations of sodium [30]. In the present study, diets were formulated to contain 1.90 g/kg sodium with a dietary electrolyte balance (DEB) of 203 mEq/kg, which may have been insufficient to maintain optimal sodium pump activity. The suggestion of Johnson and Karunajeewa [31] was that a DEB from 250 to 300 mEq/kg was optimal for young broilers, thus it may be prudent to formulate to higher sodium and DEB levels in reduced-CP diets.

**Table 11:** Linear relationships between distal jejunal apparent digestibility coefficients of starch and 16 amino acids in birds offered either wheat-based or sorghum-based diets

Amino acid	Wheat-based diets		Sorghum-based diets	
	Correlation coefficient (*r*)	Significance (*P*)	Correlation coefficient (*r*)	Significance (*P*)
Arginine	−0.536	0.007	0.408	0.048
Histidine	−0.509	0.011	0.469	0.021
Isoleucine	−0.559	0.005	0.503	0.012
Leucine	−0.567	0.004	0.527	0.008
Lysine	−0.543	0.006	0.324	0.123
Methionine	−0.274	0.195	0.361	0.084
Phenylalanine	−0.508	0.011	0.355	0.088
Threonine	−0.484	0.017	0.464	0.023
Valine	−0.562	0.004	0.501	0.013
Alanine	0.138	0.519	0.501	0.013
Aspartic acid	0.216	0.311	0.281	0.183
Glutamic acid	−0.341	0.103	0.613	0.001
Glycine	−0.656	0.001	0.496	0.014
Proline	−0.503	0.012	0.659	0.000
Serine	−0.091	0.673	0.270	0.202
Tyrosine	−0.129	0.548	0.292	0.166

Distal jejunal starch digestibility was compromised in bird offered reduced CP, wheat-based diets, but not sorghum-based diets, and this appears to be a consequence of amino acids competing with glucose for intetsinal uptakes. All ten amino acids that were negatively correlated with starch to significant extents in the wheat diets contained a proportion of non-bound entities. Overall, the proportion of non-bound amino acids of their analysed dietary concentrations averaged 45.1%, which ranged from 13.8% for proline to 60.7% for threonine. The 4 amino acids (alanine, aspartic acid, serine, tyrosine) that were present only as protein-bound entities were not significantly correlated with starch. Thus, the non-bound forms of the amino acids that were negatively correlated with starch would have been largely absorbed in the jejunum. However, the reduced-CP, sorgum-based diet also contained similar proportions of amino acids as non-bound entities. Arguably, the critical difference is the starch digestion rates between the two feed grains; the in vitro stach digestion rate of wheat (0.035 per min) is more rapid than sorghum (0.018 per min) by a factor of 1.94 [7]. Therefore, in birds offered the wheat-based diets, non-bound amino acids would have encoutered more glucose to compete with for intestinal uptakes along the jejunum than their counterparts offered sorghum-based diets.

Sorghum contains more leucine than wheat in relative and absolute terms. In one Australian survey [11], 17 sorghum samples contained 13.6 g/kg leucine or 13.3% of crude protein (101.9 g/kg). In contrast, 27 wheat samples contained 8.4 g/kg leucine or 7.3% of crude protein (115.5 g/kg). Consequently, in the present study the reduced-CP, wheat-based diet contained 4.60 g/kg non-bound leucine or 36.8% of the analysed dietary concentration of 12.5 g/kg. Alternatively, the corresponding sorghum-based diet contained 1.04 g/kg non-bound leucine, only 6.7% of the higher dietary concentration of 15.5 g/kg. These differences assume relevance when the Greenhalgh et al. [32] study is given consideration. In this study, elevated dietary concentrations of leucine in wheat- or sorghum-based reduced-CP diets (187.5 g/kg) generated contrasting growth performance responses in broiler chickens from 7 to 28 d post-hatch. Elevated leucine concentrations significantly enhanced weight gains by 9.26% in sorghum-based diets but depressed gains by 5.62% in wheat-based diets. A fractional advantage in FCR of 0.36% was observed with sorghum as opposed to a disadvantage of 1.24% with wheat. The elevated leucine, wheat-based diets in Greenhalgh et al. [32] contained 8.91 g/kg non-bound leucine, 81.7% of the analysed dietary leucine concentration of 10.9 g/kg, whereas the sorghum-based diets contained 4.75 g/kg non-bound leucine or 40.9% of the analysed dietary leucine value of 11.6 g/kg. The probability is that non-bound and protein-bound amino acids are not completely bioequivalent due to differences in digestive dynamics [33] and Metges et al. [34] made this case for leucine, specifically. Intestinal uptakes of protein-bound leucine occur slowly in broiler chickens because the amino acid is hydrophobic [35], but intestinal uptakes of NBAA are rapid as digestion is not required [36]. This raises the likelihood that the larger quantities of non-bound leucine in wheat-based diets will be subject to post-prandial oxidation [37]. Moreover, it was demonstrated in rats and humans by Nolles et al. [38] that postprandial oxidative losses were significantly higher for non-bound leucine than leucine derived from intact protein (egg white). Therefore, it seems possible that the higher proportion of protein-bound leucine in the reduced-CP sorghum-based diet than the corresponding wheat diet advantaged broiler performance.

In the present study, NBAA inclusions in the 175 g/kg CP diets based on wheat (47.4 g/kg) and sorghum (45.9 g/kg) were similarly high; however, the sorghum-based diet contained 8.83 g/kg non-bound glutamine as opposed to 1.00 g/kg in the wheat-based diet. However, glutamine plasma concentrations in birds offered 175 g/kg CP diets based on wheat or sorghum were comparable at 204 and 212 mmol/L, respectively. As analysed dietary concentrations and digestibility coefficients of glutamic acid from standard procedures do not make the distinction between glutamine and glutamate, they are not very instructive. The potential of glutamine in reduced-CP diets was reviewed by Selle et al. [39] and in a total of 20 studies 9.95 glutamine was included in 213 g/kg CP diets from 3 to 33 d post-hatch on average. The additional glutamine improved weight gain by an average of 3.25% and FCR by 2.61%. Positive weight gain responses were reported in 15 studies and 17 positive responses in FCR were recorded in the 20 studies. In addition to dietary glutamine, this amino acid is derived from the condensation of glutamate, in a reaction catalysed by glutamine synthetase, and this reaction is pivotal to both NH_3_ detoxification and glutamine biosynthesis [40]. Also, glutamine is vital for the maintenance of acid-base homeostasis and birds offered reduced-CP diets may be challenged by metabolic acidosis [41]. Thus, the additional 7.83 g/kg non-bound glutamine in the reduced-CP, sorghum-based diet may have advantaged sorghum over wheat but this is not supported by the free glutamine systemic plasma concentrations observed.

The reduction in dietary CP did not influence energy utilisation (AME, AME:GE ratios, AMEn), which was not anticipated given our consistently positive previous outcomes. However, the reduction in dietary CP did enhance N retention by 5.6 percentage units (69.7% versus 64.1%; *P* < 0.001). Interestingly, the meta-analysis completed by Alfonso-Avila et al. [42] indicated that a percentage point reduction in dietary CP content generates an average two percentage point increase in N utilization efficiency. Thus, the 5.6 percentage unit increase in N retention observed with a 30 g/kg dietary CP reduction is consistent with the meta-analysis.

Perturbations in amino acid digestibility coefficients generated by dietary CP reductions are a real obstacle to the development of reduced-CP diets. Fluctuations in amino acid digestibilities are evident in the present study and this issue is addressed more fully by Liu et al. [12]. Kafirin, the dominant protein fraction in sorghum, is poorly digestible [16] and it appears that this factor contributed to the fluctuations in amino acid digestibilities observed in the present study.

Concentrations of free amino acids in systemic plasma are difficult to interpret because they may be derived exogenously from the diet, endogenously from protein turnover and, in the case of non-essential amino acids, from biosynthesis [43]. However, it is of interest that plasma threonine concentrations were not influenced by dietary treatment in the present study. As an example, reductions in CP of maize-based diets from 200 to 172 and 156 g/kg generated increases in threonine plasma concentrations of 41.6% (715 versus 505 µmol/L) and 116.4% (1,093 versus 505 µmol/L), respectively. The implications of elevated threonine plasma concentrations in reduced-CP diets for broiler chickens have been specifically considered by Macelline et al. [44] and that increases were not observed in the present study is encouraging. Finally, it is evident in Tables 1 and 2 that the reduced-CP, sorghum-based diet contained higher lipid concentrations than the corresponding wheat-based diet. This was necessary to maintain equivalent energy densities but this would have impacted on dietary lipid:starch ratios. Increasing nutrient density has been shown to increased weight gain, decrease feed intake and improve feed conversion efficiency in broiler chickens; however, lipid had a more pronounced impact on feed intake than dietary starch concentrations [45]. Lipid concentrations and digestibilities were not determined in the present experiment, but the higher lipid concentrations and lipid:starch ratios in the reduced-CP, sorghum-based diet may have been advantageous.

## Conclusion

This study has demonstrated that birds offered sorghum-based diets have a greater capacity to accommodate dietary CP reductions than their counterparts offered wheat-based diets. It appeared that the more rapid digestion rate of wheat starch triggered competition between amino acids and glucose for intestinal uptakes along the jejunum to the detriment of starch digestibility. This may have been one of the major reasons for the outcome. If so, this emphasises the importance of the digestive dynamics of starch and protein to the growth performance of broiler chickens [46]. This study confirms that wheat-based diets are not conducive to CP reductions but without precisely identifying the more general causal factors.

## Data Availability

Additional data is available from the Corresponding Author on request.

## Abbreviations

AME: Apparent metabolizable energy
BCAA: Branched-chain amino acids
CP: Crude protein
FCR: Feed conversion ratio
N: Nitrogen
NBAA: Non-bound amino acids
NH_3_: Ammonia
